# Evaluation of treatment outcomes and associated risk factors in children with TB in Bangladesh

**DOI:** 10.5588/pha.24.0050

**Published:** 2025-03-01

**Authors:** A. Madan, S. Kulkarni, M.M. Rahman, F. Hossain, M.K. Kamul, J.I. Campbell, M.T. Rahman, J. Creswell, H. Hussain, T. Roy, A.A. Malik, M.B. Brooks

**Affiliations:** ^1^ Boston University School of Public Health, Boston, MA, USA;; ^2^ Boston Medical Center, Boston, MA, USA;; ^3^ Interactive Research & Development Bangladesh, Dhaka, Bangladesh;; ^4^ Bangladesh Shishu Hospital & Institute, Dhaka, Bangladesh;; ^5^ Innovations & Grants Team, Stop TB Partnership, Geneva, Switzerland;; ^6^ IRD Global, Singapore;; ^7^ O’Donnell School of Public Health, UT Southwestern Medical Center, Dallas, TX, USA.

**Keywords:** pediatric tuberculosis, loss to follow-up, unsuccessful treatment outcomes, LTFU

## Abstract

**BACKGROUND:**

Children aged 0–14 years old make up 4% of the total number of people diagnosed with TB in Bangladesh. Local pediatric treatment outcomes and associated factors are poorly understood; further understanding can inform tailored interventions to close delivery gaps.

**METHODS:**

To assess the risk factors for unsuccessful treatment outcomes among children receiving TB treatment in 119 health facilities in Mymensingh Division, we conducted systematic verbal screening from 2018 to 2021. Unsuccessful outcomes, including death, treatment failure, or loss to follow-up (LTFU), were analysed using log-binomial regression to examine the association with demographic and clinical characteristics.

**RESULTS:**

Among 1,967 children with reported outcomes, 99.3% (*n* = 1,954) were successful. The primary reason for unsuccessful treatment was LTFU (*n* = 12, 0.6%), followed by treatment failure (*n* = 1, 0.1%). After controlling for age and sex, children with fever had a reduced risk of unsuccessful outcomes compared to those without fever (RR 0.23, 95% CI 0.06–0.82).

**CONCLUSION:**

Most children with TB were successfully treated. LTFU was the leading reason for unsuccessful treatment outcomes in this cohort. Children with fever were less likely to have unsuccessful treatment outcomes, possibly because they were more intensely engaged in care than children without fever due to their presentation of symptoms. Continued research on pediatric TB presentation and treatment outcomes is essential for developing targeted strategies for early detection and treatment support.

Although TB is preventable and curable, it is the top cause of global deaths related to an infectious disease.^1,2^ The WHO reports that over 10 million people fall ill with TB each year, among which 12% are children <15 years old.^2^ In 2023, there were an estimated 191,000 TB-related deaths in children <15 years old.^2^ Diagnosing TB in children aged <15 years can be challenging due to non-specific symptoms, difficulty obtaining sputum specimens, and the frequency of paucibacillary disease.^3^ TB can progress quickly in children, underlining the need to initiate treatment rapidly and for monitoring treatment outcomes.^4^ Children who complete TB treatment are at lower risk of developing long-term complications, such as lung disease, and of transmitting TB to individuals in their households and communities.^5^

Bangladesh is among the 30 high-burden countries for TB, with 303,686 individuals notified with new or relapse TB and 44,170 deaths reported in 2023.^6^ Success rates of 95% have been observed for individuals with new and relapsed TB.^7^ Children aged 0–14 years old made up 4% of the 302,813 people diagnosed with a new or relapse case of TB in Bangladesh in 2023, which is lower than the 8% that children make up in the overall WHO South-East Asia Region.^7^ A recent qualitative study in Bangladesh identified caregivers’ knowledge and experience, health providers’ service provision and diagnosis, community mobilisation and case-finding efforts, and facility-level logistics issues as major challenges in identifying and diagnosing TB in children.^8^ Delays in identification and diagnosis can lead to more severe disease at diagnosis and worse treatment outcomes.

Variability in treatment outcomes between subgroups of children and factors associated with poor treatment outcomes locally is not well understood; further understanding of what drives poor outcomes in this setting can inform tailored interventions to close delivery gaps. In this study, we evaluated the treatment outcomes of children diagnosed with pulmonary TB in Bangladesh, including risk factors for unsuccessful outcomes.

## METHODS

### Study design

A prospective TB screening program was implemented programmatically in 119 facilities (41 public and 78 private) in the Mymensingh Division, Bangladesh, between November 2018 and September 2021. This work was implemented by Interactive Research & Development, Bangladesh (IRD Bangladesh) under the TB REACH Initiative (Wave 6), aiming to increase the local detection of children with TB. A multifaceted strategy to identify childhood TB includes systematic screening in clinics, household contact tracing, and the establishment of a source patient identification system within households of identified child TB patients. Invitations were extended to contacts, and efforts were made to facilitate their visits to health facilities. In individuals where attendance was not possible, project staff conducted home visits. Verbal screenings were systematically conducted during these interactions, accompanied by complimentary TB tests. To bolster community awareness, diverse communication channels were employed in collaboration with government initiatives. This included the distribution of approved child TB communication materials—such as 100,000 leaflets through newspaper inserts and community engagement programs, alongside 10,000 child TB awareness posters strategically placed across various locations, including community clinics, private providers chambers, hospitals, public gathering places, and government offices.

### Study population

The study population included all children (0–14 years old) admitted to inpatient departments and those visiting pediatric outpatient departments across the 119 participating facilities who were diagnosed with pulmonary TB. Details are reported elsewhere.^9^

### Key procedures and measures

All children were verbally screened for TB symptoms or recent contact with an individual sick with TB disease by program staff. If presumed to have TB based on any reported symptoms or contact history, children were referred for a clinical evaluation, chest X-ray, and TB diagnostic testing. When children were diagnosed, they were initiated on treatment per national guidelines and followed throughout treatment until an outcome was observed.

The primary outcome measure was the occurrence of an unsuccessful treatment outcome, defined as loss to follow-up (LTFU), treatment failure, or death among children diagnosed with TB. Predictors explored included demographic characteristics (age, sex), clinical signs and symptoms (weight under the 5^th^ percentile for age, cough and cough duration, fever lasting longer than 2 weeks, night sweats, any swelling or nodule in the body, weight loss, decreased appetite), presence of a bacille Calmette-Guérin scar, TB contact history, and reporting a family member with TB. We tried to include all characteristics and symptoms associated with TB and previous poor treatment outcomes.

### Analysis

All children diagnosed with pulmonary TB during the screening program and who subsequently initiated TB treatment were considered eligible for inclusion in the analysis. Children whose treatment outcome was reported as ‘not evaluable’ during data collection were excluded from the analysis. The frequency of characteristics among children diagnosed with TB was reported, and chi-squared tests were used to examine the distribution of characteristics across age groups (0–4, 5–9, 10–14 years). We report detailed characteristics of children who died before treatment initiation. Furthermore, we report treatment outcomes of children who initiated TB treatment and had an evaluable treatment outcome. We then report detailed characteristics of children who experienced an unsuccessful treatment outcome. Univariable log-binomial regression was used to estimate the risk ratios for the association of patient characteristics with experiencing an unsuccessful treatment outcome. Any characteristic identified to have an association with experiencing an unsuccessful treatment outcome on univariable analysis (*p* < 0.20) was considered for inclusion in the multivariable model; variables with a significance level of *p* < 0.05 were maintained in the final multivariable model, controlling for patient age group and gender. Data were analysed using SAS Studio v9.4 (SAS, Cary, NC, USA).

### Ethics approval

The Institutional Review Board (IRB) of BRAC James P Grant School of Public Health, BRAC University, Dhaka, Bangladesh, reviewed and approved the original study protocol (approval number: 2019-037-ER). Verbal informed consent was obtained from all children’s guardians and children over the age of 7 years. The subsequent analysis of de-identifiable data was determined to be non-human subjects research by the IRB of Boston University Medical Campus, Boston, MA, USA (H-43034).

## RESULTS

Between November 2018 and September 2021, a total of 2,189 children were diagnosed with TB in this screening programme. There were 2,001 (91.4%) children diagnosed with pulmonary TB. A total of 322 (16.1%) children were 0–4 years old, 738 (36.9%) children were 5–9 years old, and 941 (47.0%) children were 10–14 years old (Table 1). Less than half of all children were female (*n* = 929, 46.4%), with lower percentages in the 0–4 and 5–9-year-old groups (*n* = 137, 42.6%; and *n* = 311, 42.1%, respectively) than the 10–14-year-old group (*n* = 481, 51.1%; *p* < 0.001). A majority of children (*n* = 1,262, 63.0%) had weight less than the 5^th^ percentile for age, although this decreased as the age group increased: 233 (72.4%), 483 (65.5%), and 546 (58.0%) in children 0–4, 5–9, and 10–14 years old, respectively (*p* < 0.001).

**TABLE 1.** Characteristics of children diagnosed with pulmonary TB disease in Mymensingh Division, Bangladesh, stratified by age group (*n =* 2,001).

Characteristic	Total (*n* = 2,001) *n* (%)	Children aged 0–4 years (*n* = 322) *n* (%)	Children aged 5–9 years (*n* = 738) *n* (%)	Children aged 10–14 years (*n* = 941) *n* (%)	*p*-value
Demographics					
Age, years, median [IQR]	9 [6–12]	2 [1–^‡^]	7 [6–8]	13 [11–14]	n/a
Age groups, years					
0–4	322 (16.1)	322 (100)	n/a	n/a	n/a
5–9	738 (36.9)	n/a	738 (100)	n/a	n/a
10–14	941 (47.0)	n/a	n/a	941 (100)	n/a
Female	929 (46.4)	137 (42.6)	311 (42.1)	481 (51.1)	<0.001
Symptoms, clinical presentation, medical history					
Weight <5^th^ percentile	1,262 (63.0)	233 (72.4)	483 (65.5)	546 (58.0)	<0.001
Any cough	1,722/1,994 (86.4)	281/320 (87.8)	621/737 (84.3)	820/937 (87.5)	0.111
Cough >2 weeks	(*n* = 1,721) 1,457 (84.6)	(*n* = 281) 248 (88.3)	(*n* = 620) 512 (82.6)	(*n* = 820) 697 (85.0)	0.085
Fever >2 weeks	1,869/1,999 (93.5)	297 (92.2)	687/737 (93.2)	885/940 (94.2)	0.451
Night sweats	1,189/1,978 (60.1)	189/320 (59.1)	422/725 (58.2)	578/933 (62.0)	0.278
Swelling	463/1979 (23.4)	61/318 (19.2)	191/727 (26.3)	211/934 (22.6)	0.035
Weight loss	1,791/1,998 (89.6)	271 (84.2)	666/736 (90.5)	854/940 (90.9)	0.002
Appetite decrease	1,738/1,996 (87.1)	255 (79.2)	648/734 (88.3)	835/940 (88.8)	<0.001
BCG scar	1,961/1,983 (98.9)	310/318 (97.5)	726/734 (98.9)	925/931 (99.4)	0.023
Contact history	1,513/1,991 (76.0)	237 (73.6)	589/731 (80.6)	687/938 (73.2)	0.001
Family smoking	1,721 (86.0)	271 (84.2)	633 (85.8)	817 (86.82)	0.481
Family history of TB					
Family member with TB?	1,512/1,975 (76.6)	234/317 (73.8)	590/728 (81.0)	688/930 (73.8)	0.002
Which family member?	(*n* = 1,512)	(*n* = 234)	(*n* = 590)	(*n* = 688)	0.113
Mother	515 (34.1)	72 (30.8)	200 (34.0)	243 (35.3)	
Father	268 (17.7)	49 (21.0)	95 (16.1)	124 (18.0)	
Sibling	126 (8.3)	17 (7.3)	47 (8.0)	62 (9.0)	
Grandparent	267 (17.7)	58 (24.8)	105 (17.8)	104 (15.1)	
Other	336 (22.2)	38 (16.2)	143 (24.2)	155 (22.5)	
Type of TB disease					
Bacteriologic confirmation of disease	144 (7.2)	17 (5.3)	30 (4.1)	97 (10.3)	<0.001

IQR = interquartile range; n/a = not available; BCG = bacille Calmette-Guérin.

The most frequently reported symptoms were cough (1,722; 86.4%), among which 1,457 (84.6%) reported cough for over 2 weeks duration, fever (1,869; 93.5%), weight loss (1,791; 89.6%), and decreased appetite (1,738; 87.1%). The frequency of some reported symptoms varied significantly by age group, with younger children reporting less weight loss (*p* = 0.002) and decreased appetite (*p* < 0.001) than older children. Furthermore, a history of TB contact was reported among 1,513 (76.0%) children, with the highest percentage reported among the 5–9-year-old groups (*p* < 0.001). Only 144 (7.2%) children had bacteriologic confirmation of disease, with the percentage increasing with age group (*p* < 0.001).

Four (0.2%) children died prior to treatment initiation; 3 (75.0%) were aged ≤3 years, and 3 (75.0%) were males (Table 2). All 4 children reported having a fever lasting longer than 2 weeks, and all 3 children with a response reported cough for over 2 weeks. Notably, none of these children had documented contact with a family member with TB.

**TABLE 2.** Detailed characteristics of the children who died before TB treatment initiation (*n* = 4).

#	Age years	Gender	Weight <5^th^ percentile %	Cough >2 weeks	Fever >2 weeks	Night sweats	Swelling	Weight loss	Decreased appetite	BCG scar	Family contact history
1	0	F	N	Y	Y	N	Y	Y	Y	Y	N
2	0	M	Y	Y	Y	N	N	N	N	Y	N
3	3	M	Y	Y	Y	Y	N	N	N	Y	N
4	13	M	N	—	Y	N	N	Y	Y	Y	N

BCG = bacille Calmette-Guérin; F = female; M = male; N = no; Y = yes.

Of the 1,997 children who initiated TB treatment, 30 (1.5%) did not have an evaluable treatment outcome at the time of data collection completion, so they were excluded from further analysis. Characteristics of these 30 children were similar to those reported in the overall cohort (Table 3). Of the remaining 1,967 children, 1,958 (99.3%) had a successful outcome, while 13 (0.7%) experienced an unsuccessful outcome; 12 (0.6%) were lost to follow-up and 1 (0.1%) experienced treatment failure (Table 4). These outcomes did not vary significantly by age group (*p* = 0.765). Amongst children with bacteriologic confirmation of disease and an evaluable treatment outcome (*n* = 141), all children had a successful cure outcome.

**TABLE 3.** Descriptive statistics for children initiated on TB treatment whose treatment outcomes were not evaluable.

#	Age year	Sex	Weight <5^th^ %	Cough >2 weeks	Fever >2 weeks	Night sweats	Swelling	Weight loss	Decreased appetite	BCG scar	Family contact history	Family member with TB
1	0	F	Y	Y	Y	N	N	Y	Y	Y	N	—
2	1	M	Y	Y	Y	N	N	Y	Y	Y	Y	Mother
3	3	M	Y	—	Y	N	Y	N	Y	Y	N	—
4	5	F	Y	Y	Y	N	N	Y	Y	Y	N	—
5	5	F	Y	Y	Y	Y	N	Y	N	Y	N	—
6	6	M	Y	Y	Y	Y	N	Y	Y	Y	Y	Mother
7	7	M	Y	Y	Y	Y	N	Y	Y	Y	N	—
8	7	F	N	Y	Y	N	N	N	Y	Y	Y	Mother
9	7	M	Y	Y	Y	N	Y	Y	Y	Y	Y	Other
10	8	F	Y	Y	Y	Y	N	Y	Y	Y	Y	—
11	9	F	N	Y	Y	Y	N	Y	Y	Y	Y	—
12	9	M	N	Y	Y	Y	N	Y	Y	Y	Y	Mother
13	9	F	N	—	Y	N	Y	Y	Y	Y	Y	Other
14	9	F	N	Y	Y	Y	Y	Y	Y	Y	Y	Mother
15	9	F	Y	N	N	Y	N	N	Y	N	Y	Other
16	10	F	Y	Y	Y	Y	N	Y	Y	Y	Y	Mother
17	10	M	N	Y	Y	Y	N	Y	Y	Y	Y	Mother
18	10	M	Y	Y	Y	Y	N	Y	Y	Y	Y	Father
19	10	F	Y	N	N	Y	Y	N	Y	Y	Y	Other
20	10	F	Y	N	N	Y	Y	N	Y	Y	Y	Mother
21	10	M	Y	Y	Y	Y	N	Y	Y	Y	Y	Grandparent
22	11	F	Y	Y	Y	N	N	N	N	Y	Y	Other
23	11	F	N	Y	Y	Y	N	Y	Y	Y	Y	Mother
24	12	M	N	Y	Y	N	N	Y	Y	Y	N	—
25	13	M	Y	Y	Y	Y	N	Y	Y	Y	Y	Mother
26	13	F	Y	Y	Y	Y	N	Y	Y	Y	Y	Mother
27	14	F	N	Y	Y	N	N	N	N	Y	Y	Sibling
28	14	F	N	Y	Y	N	N	Y	Y	Y	Y	Father
29	14	M	N	Y	Y	Y	N	Y	Y	Y	Y	Other
30	14	M	N	Y	Y	Y	N	Y	Y	Y	Y	Other

BCG = bacille Calmette-Guérin; F = female; M = male; N = no; Y = yes.

**TABLE 4.** Treatment outcomes of 1,967 children who initiated TB treatment and had an evaluable outcome.

Treatment outcome	Total (*n* = 1,967) *n* (%)	Children aged 0–4 years (*n* = 316) *n* (%)	Children aged 5–9 years (*n* = 726) *n* (%)	Children aged 10–14 years (*n* = 925) *n* (%)
Successful	1,954 (99.3)	313 (99.1)	722 (99.4)	919 (99.3)
Cure	141 (7.2)	17 (5.3)	30 (4.1)	94 (10.0)
Treatment completed	1817 (92.2)	296 (92.8)	692 (95.3)	825 (89.2)
Unsuccessful	13 (0.7)	3 (0.9)	4 (0.6)	6 (0.7)
Died	0 (0)	0 (0)	0 (0)	0 (0)
Treatment failure	1 (0.1)	0 (0)	0 (0)	1 (0.1)
Loss to follow-up	12 (0.6)	3 (0.9)	4 (0.5)	5 (0.5)

In the 13 children with unsuccessful treatment outcomes, 6 (46.2%) were in the 10–14-year-old age group, 7 (53.8%) were female, 8 (61.5%) had weight below the 5th percentile for age, 7 (53.8%) had cough lasting longer than 2 weeks, 10 (76.9%) presented with fever lasting longer than 2 weeks, 9 (69.2%) had night sweats, 10 (76.9%) reported weight loss, and 10 (76.9%) reported decreased appetite (Table 5). In the 12 (92.4%) instances in which the child had a family member with TB, the family member was a parent for half of them (*n* = 6, 50.0%).

**TABLE 5.** Demographic and clinical characteristics of 13 children who had an unsuccessful TB treatment outcome.

#	Age years	Gender	Weight <5^th^ percentile %	Cough >2 weeks	Fever >2 weeks	Night sweats	Swelling	Weight loss	Decreased appetite	BCG scar	Family contact history	Family member with TB	Reason for poor treatment outcome
1	0	F	Y	Y	Y	N	N	Y	N	Y	N	—	LTFU
2	1	F	Y	Y	Y	Y	N	Y	Y	Y	Y	Mother	LTFU
3	3	M	Y	N	N	N	Y	N	N	Y	Y	Grandparent	LTFU
4	6	M	Y	N	Y	Y	N	Y	Y	Y	Y	Mother	LTFU
5	7	F	N	N	N	Y	N	N	Y	Y	Y	Other	LTFU
6	7	M	N	Y	Y	Y	N	Y	Y	Y	Y	Mother	LTFU
7	8	M	Y	Y	Y	Y	N	Y	Y	Y	Y	Father	LTFU
8	10	F	Y	—	N	Y	Y	Y	Y	Y	Y	Other	Treatment failure
9	10	M	N	Y	Y	Y	N	Y	Y	Y	Y	Father	LTFU
10	10	F	N	Y	Y	Y	N	Y	Y	Y	Y	Grandparent	LTFU
11	12	F	N	—	Y	N	Y	N	N	Y	Y	Mother	LTFU
12	14	F	Y	Y	Y	N	N	Y	Y	Y	Y	Grandparent	LTFU
13	14	M	Y	N	Y	Y	N	Y	Y	Y	Y	Sibling	LTFU

BCG = bacille Calmette-Guérin; F = female; LTFU= loss to follow-up; M = male; N = no; Y = yes.

In univariable analysis, children with fever for over 2 weeks, compared to those without, had a decreased risk of experiencing an unsuccessful treatment outcome (relative risk [RR] 0.23, 95% confidence interval [CI] 0.06–0.82). After controlling for both age group and gender, the association of fever with unsuccessful treatment outcome did not change (Table 6).

**TABLE 6.** Univariable and multivariable analyses assessing risk factors for children with TB experiencing an unsuccessful treatment outcome.*

	Univariable RR (95% CI)	*p*-value	Multivariable aRR (95% CI)	*p*-value
Age group, years				
0–4	Reference	Reference	Reference	Reference
5–9	0.58 (0.13–2.60)	0.48	0.59 (0.13–2.60)	0.50
10–14	0.68 (0.17–2.70)	0.59	0.70 (0.17–2.80)	0.61
Female sex	1.35 (0.46, 4.00)	0.59	1.4 (0.48–4.20)	0.52
Weight <5^th^ percentile	0.93 (0.31–2.80)	0.90		
Any cough	0.87 (0.19–3.90)	0.86		
Cough >2 weeks	0.34 (0.07–1.80)	0.20		
Fever >2 weeks	0.23 (0.06–0.82)	0.02	0.23 (0.06–0.82)	0.02
Night sweats	1.5 (0.46–4.80)	0.51		
Swelling	0.98 (0.27–3.50)	0.98		
Weight loss	0.37 (0.10–1.30)	0.13		
Appetite decrease	0.49 (0.14–1.80	0.28		
Contact history	3.8 (0.49–29.0)	0.20		
Family member with TB	3.6 (0.47–28.0)	0.22		

* RR for BCG scar could not be estimated because all children experiencing an unsuccessful treatment outcome had a BCG scar present. RR for bacteriologic confirmation of disease could not be estimated because all children with bacteriologic confirmation of disease experienced a successful treatment outcome.

RR= relative risk; CI = confidence interval; aRR = adjusted RR; IQR= interquartile range; BCG = bacille Calmette-Guérin.

## DISCUSSION

Our treatment cohort had overwhelmingly positive treatment outcomes, with 99.3% of children achieving successful results. These are higher than the outcomes reported in other studies. For example, a study in Lima, Peru, reported a poor treatment outcome in 13.6% of cases, with 12.9% being lost to follow-up,^10^ while two cohort studies from Pakistan found that 4.8% of children had an unsuccessful outcome, with 2.4% being lost to follow-up^11^ and 11.8% of children having an unsuccessful outcome, with 9.3% being lost to follow-up.^12^ In contrast, the Bangladesh cohort presented here had only a 0.7% rate of unsuccessful outcomes, and 0.6% were lost to follow-up, excluding the children who died prior to TB treatment initiation. The lower rates of unsuccessful outcomes in Bangladesh may reflect the differences in healthcare systems, intervention effectiveness, attitudes toward treatment adherence, or other regional factors like the involvement of local authorities and organisations.^13^ These findings suggest that context-specific factors such as local health infrastructure, the Bangladesh National TB Programme, access to care, and community engagement strategies may play a role in ensuring higher treatment success rates.

Of the 13 children with reported poor outcomes in our cohort, 92% were lost to follow-up. Our findings align with previous studies in Botswana,^14^ Denmark,^15^ Pakistan,^11,12^ Peru,^10^ Ghana,^16^ Uganda^17^ and Zimbabwe^18^ that reported significant rates of LTFU in child TB patients. A recent systematic review found that most LTFU during TB treatment in adults (≥18 years) occurs within the first 2 months of initiating treatment.^19^ The timing of LTFU in children is not well established, but a study from Peru found that 30% of children were LTFU within 3 months of initiating treatment.^10^ Addressing the underlying causes of LTFU—whether through more intensive follow-up protocols, caregiver education, or logistical support for families—could substantially improve pediatric TB outcomes. Developing targeted interventions focusing on early recognition of individuals at high risk of LTFU and providing necessary support and follow-up could help improve overall treatment success rates.

Our analysis revealed that a fever lasting longer than 2 weeks at baseline was protective against experiencing an unsuccessful treatment outcome. This association held even after adjusting for age and sex. Other studies have identified persistent fever as a significant predictor of poor treatment outcomes in TB; one study in Pakistan identified an increased risk of experiencing an unsuccessful treatment outcome in children presenting with fever (RR 2.56, 95% CI 1.02–6.44), although in this study only half (50%) of children experiencing an unsuccessful treatment outcome were lost to follow-up, while 29% had treatment failure and 21% died.^11^ If it continues despite treatment, fever may signal more severe TB manifestations or complications such as drug resistance.^20^ Our findings suggest that considering prolonged fever as a marker of disease severity should also include caregivers’ perceptions of the child’s health, resulting in more focus on febrile children and increased resource prioritisation. This may lead to more attention to care and better treatment adherence. Further research with larger sample sizes is necessary to explore whether these findings are significant in other settings or larger populations.

Other characteristics*,* such as weight being equal to or lower than the 5^th^ percentile, night sweats, contact history, and family members with TB, were not significant drivers of poor treatment outcomes. One possible explanation could be the small number of children with a poor outcome.

Thirty children in our cohort did not have evaluable health outcomes and were excluded from the analysis. Some of these children may have completed their treatment and may not have returned to the clinic at the time of final evaluation for a treatment outcome to be declared, as was seen in previous work.^10^ Because we did not have information on treatment duration or adherence, we were unable to ascertain this possibility and thus excluded these children from the analysis. We did not perform a sensitivity analysis considering these children as treatment completed and LTFU; those analyses may have led to ambiguous and erroneous associations because the number of patients with unevaluable outcomes was much larger than the number with known poor treatment outcomes. For further reference, we provide clinical information on these children in Table 3.

Limitations of our study include an inability to determine the exact timing of when children were lost to follow-up. This prevents developing an understanding of at what point in treatment these children were lost and the barriers to care that may have contributed to LTFU for these children. Furthermore, the small number of children with poor treatment outcomes restricts our ability to detect other statistical associations between clinical symptoms and adverse outcomes. Additionally, we did not have data on other known TB risk factors, such as HIV status. Despite these limitations, the large sample size provides a strong representation of the pediatric population in Bangladesh and lends weight to the generalizability of our findings.

Our study findings have implications for policy and clinical practice in pediatric TB management. Given the high treatment success rate but notable challenge of LTFU, policies must prioritize strengthening follow-up mechanisms, especially in resource-limited settings like Bangladesh. Programs could implement community-based health worker models that track and support pediatric patients during treatment. Children with fever were less likely to have unsuccessful treatment outcomes, possibly because they were more intensely engaged in care than children without fevers due to their presentation of symptoms. Continued research on pediatric TB presentation and treatment outcomes is essential for developing targeted strategies for early detection and treatment support. This study can help identify areas in which future research should be conducted to understand better the root cause of programmatic gaps in care, as well as areas that were successful in developing strategies for sustainability. Additionally, future studies should investigate specific reasons for LTFU and explore social, economic, and behavioural barriers to treatment adherence. Policymakers should advocate for these integrated approaches, ensuring that pediatric TB management encompasses medical treatment and broader social determinants of health.
